# Muscle contributions to medial tibiofemoral compartment contact loading following ACL reconstruction using semitendinosus and gracilis tendon grafts

**DOI:** 10.1371/journal.pone.0176016

**Published:** 2017-04-19

**Authors:** Jason M. Konrath, David J. Saxby, Bryce A. Killen, Claudio Pizzolato, Christopher J. Vertullo, Rod S. Barrett, David G. Lloyd

**Affiliations:** 1 School of Allied Health Sciences and Menzies Health Institute Queensland, Griffith University, Gold Coast, Queensland, Australia; 2 Knee Research Australia, Gold Coast, Queensland, Australia; Northwestern University Feinberg School of Medicine, UNITED STATES

## Abstract

**Background:**

The muscle-tendon properties of the semitendinosus (ST) and gracilis (GR) are substantially altered following tendon harvest for the purpose of anterior cruciate ligament reconstruction (ACLR). This study adopted a musculoskeletal modelling approach to determine how the changes to the ST and GR muscle-tendon properties alter their contribution to medial compartment contact loading within the tibiofemoral joint in post ACLR patients, and the extent to which other muscles compensate under the same external loading conditions during walking, running and sidestep cutting.

**Materials and methods:**

Motion capture and electromyography (EMG) data from 16 lower extremity muscles were acquired during walking, running and cutting in 25 participants that had undergone an ACLR using a quadruple (ST+GR) hamstring auto-graft. An EMG-driven musculoskeletal model was used to estimate the medial compartment contact loads during the stance phase of each gait task. An adjusted model was then created by altering muscle-tendon properties for the ST and GR to reflect their reported changes following ACLR. Parameters for the other muscles in the model were calibrated to match the experimental joint moments.

**Results:**

The medial compartment contact loads for the standard and adjusted models were similar. The combined contributions of ST and GR to medial compartment contact load in the adjusted model were reduced by 26%, 17% and 17% during walking, running and cutting, respectively. These deficits were balanced by increases in the contribution made by the semimembranosus muscle of 33% and 22% during running and cutting, respectively.

**Conclusion:**

Alterations to the ST and GR muscle-tendon properties in ACLR patients resulted in reduced contribution to medial compartment contact loads during gait tasks, for which the semimembranosus muscle can compensate.

## Introduction

The quadruple bundle hamstring graft using the semitendinosus (ST) and gracilis (GR) tendons has become an increasingly common orthopaedic technique for anterior cruciate ligament (ACL) reconstruction (ACLR). The graft possesses excellent material strength [1] and has minimal impact on the knee extensor mechanism [2–4]. However, following harvest, the size of the donor muscles are substantially reduced [5] resulting in knee flexor and internal rotation weakness [6–8]. Although there is some evidence of compensatory hypertrophy of the other hamstring muscles [6], the loss of muscle size in ST and GR likely compromises their force producing capability. This could in turn, have implications for tibiofemoral joint function, stability and loading.

During gait, the muscles that span the tibiofemoral joint play a critical role in forward propulsion, frontal plane tibiofemoral stability and contact loading [9, 10]. A muscle’s contribution to medial compartment contact loading is strongly associated with its capacity to stabilise external valgus moments, whilst a muscle’s contribution to lateral compartment loading is associated with its capacity to stabilise external varus moments [10]. Since the ST and GR are common donor muscles used for replacement of the injured ACL and have large moment arms capable of stabilising external valgus loads [11], the loss of ST and GR muscle size may reduce their contribution to both medial compartment contact loading and the stability of the tibiofemoral joint. Previous studies have found that the peak knee adduction moment is related to disease severity [12, 13], however, given the substantial contributions made by muscles to the contact loading of the knees articular surfaces [9, 10], and their mechanical role in stabilising the joint against external loads, methods that estimate knee joint contact loads should include the contribution of the surrounding muscles.

There is emerging evidence that contact loading of the tibiofemoral joint is lower than normal following ACL rupture [14] and subsequent reconstruction [15] and is associated with future onset of knee osteoarthritis (OA) [15]. Knee OA typically affects the medial compartment, with the loss of medial cartilage being an important structural marker of disease severity and progression [16, 17]. The magnitude of the tibiofemoral joint contact force may be influenced by external loading conditions, kinematics, as well as an individuals task-specific muscle activation patterns. When compared to healthy controls, ACL reconstructed patients have been reported to walk with smaller knee flexion angles and knee flexion excursion during gait [18]. Moreover, studies comparing tibiofemoral motion and loading between the ACLRs and controls have reported both the injured and contralateral sides have significant differences compared to healthy intact knees [19]. Furthermore, Gardinier et al. [14] investigated tibiofemoral contact forces in athletes with acute ACL rupture and found that patients walked with decreased joint contact force on their injured knee compared to their uninjured knee, which persists after ACLR [15]. However no previous literature, has attempted to investigate the effect of donor muscle atrophy on their contribution to the joint contact force. In order to isolate the effects of different donor muscle-tendon properties, a comparison is needed under the same external loading conditions, kinematics and underlying muscle activation patterns that pertain to each individual.

Direct in vivo measurement of joint contact forces is only possible through the use of instrumented prosthetic implants. However, due to cost and invasiveness, direct measurement is unfeasible. An alternative approach is computational neuromusculoskeletal (NMS) models that provide a non-invasive method to estimate the tibiofemoral joint contact forces that occur during gait. Computational methods may be broadly categorised as either optimization-based or electromyography (EMG) driven models. A limitation of optimization-based models is that the assumption that the nervous system recruits muscles based on a known criterion (i.e. minimization of muscle stresses) may not apply to individuals with joint pathology or neurological impairment [20]. EMG-driven models address this shortcoming by using measured muscle activation patterns as additional model inputs [21]. Muscle activation patterns together with muscle-tendon kinematics are then used as inputs to a Hill-type muscle model to derive estimates of muscle-tendon forces and moments, as well as joint contact forces. Importantly, EMG-driven model estimates of tibiofemoral contact forces have been validated against direct measurements from instrumented knee implants [20, 22, 23].

The purpose of this study was to use a neuromusculoskeletal modelling approach to determine the effects of previously reported alterations in the muscle-tendon properties of the ST and GR in ACLR [5, 6]; on their contributions to medial compartment contact loading within the tibiofemoral joint experienced under the same motion and external loading conditions, during walking, running and sidestep cutting. We hypothesised that the ST and GR would contribute less to medial compartment loading of the tibiofemoral joint following ACLR, and that other non-donor muscles would compensate for these reductions. Since the donor muscles are involved in supporting several degrees of freedom, it was envisaged that estimating these theoretical compensation strategies would inform rehabilitation strategies in individuals that have undergone a quadruple bundle hamstring auto-graft ACLR.

## Materials and methods

### Participants

Twenty-five participants (20 male, 5 female, mean age 31 ± 6 years, mean body mass 84 ± 13kg) that had undergone a quadruple bundle hamstring (ST+GR) auto-graft ACLR were recruited. Inclusion criteria were: (i) unilateral ACL injury sustained without any concomitant knee ligament injury; (ii) between 2–3 years post a quadrupled ST-GR graft -ACLR; (iii) between 18–45 years of age; (iv) the ability to comply with testing protocol. Exclusion criteria were: (i) complex knee injuries with additional ligament tears; (ii) previous or subsequent ACL injury or lower extremity surgery. Ethics approval was obtained through Human Research Ethics Committee of the University of Western Australia (Reference Code: RA/4/1/4150) with all participants providing their written informed consent prior to any testing.

### Surgical procedure

Patients were recruited from the clinics of four local orthopaedic surgeons. Surgeons followed a standardised protocol for a quadruple bundle hamstring auto-graft. Following tourniquet application to the thigh, an anteromedial vertical incision was made over the pes anserinus. The superior border of the pes anserinus was then incised to visualise the ST and GR tendons. The tendons were left secured to their distal attachment points and an open-ended tendon harvester (Linvatec, Largo FL) was used to release the tendons proximally from their muscular attachment points using a cut rather than a push technique [24] to a length of 22 cm in females and 24 cm in males. Then a quadrupled graft was formed by folding both tendons and wound together. The femoral tunnel was created via a transportal drilling technique, with femoral fixation of the graft achieved by a closed loop Endobutton (Smith & Nephew, Memphis TN) and tibial fixation achieved using a round cannulated interference screw (Smith & Nephew, Memphis TN). Following surgery, all patients followed a standardised early mobilization rehabilitation protocol [6].

### Experimental protocol

Participants initially performed a series of maximal vertical jumps, isometric contractions, as well as isokinetic dynamometer trials in order to obtain maximum EMG values for each instrumented muscle. Participants were then familiarized with each gait task (walk, run and sidestep cut) and subsequently performed a minimum of three successful trials of each gait task. A trial was considered successful if the relevant foot landed wholly on the force platform and was performed at the desired speeds of 2.0–2.5 m/s for walking and 4–4.5 m/s for running and sidestep cutting. The sidestep cutting was performed, using the surgical leg as the pivot leg, to an angle of 45° from the approach direction.

### Experimental data collection

Motion capture, force plate and EMG data were concurrently and synchronously acquired during the performance of each task. A 10-camera VICON MX motion analysis system (Vicon, Oxford, UK) was used to acquire the motion of retro-reflective skin-surface markers attached to the participants, and sampled at 200 Hz. Retro-reflective skin-surface markers were placed on prominent anatomical landmarks in accordance with the UWA marker set [25], with 3-marker clusters attached to the upper-limb, and 10-marker clusters used on lower-limb segments to improve assessment of knee motion [26]. Ground reaction forces (GRF) were measured from two force plates (Advanced Mechanical technology Inc., Watertown, USA) sampling at 1000 Hz. EMGs from 16 muscles on the surgical limb were sampled at 1000 Hz using wireless sensors (Zerowire, Aurion, Milan, IT) bipolar Ag/AgCl surface electrodes (Duo-Trode, Myotronics, USA). The muscles investigated were: medial hamstring group (semimembranosus (SM)/semitendinosus (ST)); lateral hamstring group (biceps femoris long head (BFLH) and biceps femoris short head (BFSH)); adductor group (AG); rectus femoris (RF); vastus lateralis (VL); vastus medialis (VM); gracilis (GR); tensor fascia latae (TFL); sartorious (SR); gluteus maximus (GMax); gluteus medius (GMed); medial gastrocnemius (MG); lateral gastrocnemius (LG); soleus (SL); tibialis anterior (TA); and peroneals (PR).

### Experimental data processing

Data processing was performed using the MOtoNMS software [27] in MATLAB (The Mathworks, Mass, USA). Marker trajectories and GRFs were low-pass filtered using a zero-lag, 2^nd^ order, Butterworth filter with a cut-off frequency of 10 Hz for walking and 15 Hz for running and cutting. Static [28] and functional [25] tasks were performed to identify joint centres. EMGs were band-pass filtered (30–500 Hz), full wave rectified and then low pass filtered with a cut off frequency of 6 Hz to yield linear envelopes for each muscle [21], and subsequently normalised to their maximum value identified across all dynamic trials, functional tasks and dynamometer trials to represent the activations of 34 musculotendinous units (MTU)[29]. Knee joint centres were defined using mean helical axes [25], the hip joint centres were defined using Harrington regression [30], and the ankle joint centre was defined as the midpoint between medial and lateral malleoli [31].

### The standard model

In order to isolate the effects of different donor muscle-tendon properties under the same external loading conditions, kinematics and underlying muscle activation patterns, we chose to use the surgical leg with unadjusted muscle parameters as the standard model. The standard model was used to compute estimates of the tibiofemoral contact loads during the stance phase of each task assuming no morbidity to the ST and GR using the EMG-driven mode of the software CEINMS [32]. CEINMS has been described in detail previously [32] and so will only be described in brief here. The model consisted of four components: an anatomical model created using OpenSim [33] that contained the insertion points and paths of the line segment representation of 34 musculotendinous units (MTU), an EMG to activation model that estimated the activation of the MTUs using a second order discrete non-linear model [21], a modified Hill-type muscle model that used MTU activation and kinematics to estimate MTU forces and moments, and a calibration phase. Each MTU was modelled as a contractile element in series with a compliant tendon [34]. The tendon was modelled using a non-linear function normalised to tendon slack length ($l_{t}^{s}$) [34]. The contractile element model consists of generic force-length, force-velocity, and parallel elastic functions, in which final MTU force (*F*_*MTU*_), is dependent on each MTU’s maximum isometric force ($F_{m}^{MAX}$), optimal fibre length ($l_{m}^{o}$), and pennation angle at optimal fibre length ($\emptyset_{m}^{o}$).

Calibration was used to optimise the MTU and activation parameters for each subject. Calibration consisted of two steps: morphometric and functional scaling [29, 32, 35, 36]. The morphometric scaling adjusted the parameters of ($l_{m}^{o}$) and ($l_{t}^{s}$) of each MTU to preserve the dimensionless muscle fibre and tendon operating curves while respecting the overall MTU length across a range of lower-limb joint angles [35, 36]. The functional scaling, part of the CEINMS framework, adjusted EMG-driven model parameters such that the least squared differences between the model predicted joint moments and the experimentally measured joint moments were minimised [21, 29]. The calibration included joint moments from hip adduction-abduction (HAA), hip flexion/extension (HFE), knee flexion/extension (KFE), and ankle dorsi/plantar flexion (AFE) [29]. The experimental trials used in the calibration procedure included one walk, one run and one cut. Parameters included in the functional calibration were: (i) activation parameters (C1 and C2) which adjust the impulse response of the second order filter (ii) a non-linear shape factor (A) which accounts for the non-linear EMG to force relationship [21], (iii) $l_{m}^{o}$, (iv) $l_{t}^{s}$, and (v) strength coefficients for 12 groups of muscles that scale each MTUs $F_{m}^{MAX}$ within each group to account for differences in muscle physiological cross sectional area between people [21, 29]. The 12 functional muscle groups were the uniarticular hip flexors, uniarticular hip extensors, uniarticular hip adductors, biarticular hip adductors, hip abductors, uniarticular knee flexors, uniarticular knee extensors, uniarticular ankle plantar flexors, uniarticular ankle dorsi flexors, biarticular quadriceps, biarticular hamstrings and gastrocnemius muscles. After calibration, the NMS model operated as an *open-loop predictive system* for each of the walking, running and cutting trials to calculate muscle forces, joint moments and knee joint contact forces as a function of muscle activation and model kinematics [32].

### The adjusted model

A version of the standard model with modifications to the Hill-type muscle-model parameters for the ST and GR was created to represent donor muscle morbidity following a hamstring graft ACLR [5, 6]. Muscle volumes (*V*_*m*_) and peak cross sectional areas (*CSA*_*m*_) from Williams et al [5] and Konrath et al [6] were chosen to represent ST and GR morbidity, because both *V*_*m*_ and *CSA*_*m*_ were reported. Although Williams [5] was 6–9 months post-surgery and Konrath [6] was 2 years post-surgery, their values were similar. Therefore, these morphological changes were pooled together and used to adjust the ST and GR parameters (Table 1). The CSA of the ST and GR were reduced in the surgical leg of ACLR patients relative to the contralateral leg by 33% and 39%, respectively, and the corresponding muscle volumes were reduced by 47% and 35%, respectively (n = 28).

**Table 1:** Morphological changes to the ST and GR following ACLR.

	CSA (cm^2^)		Volume (cm^3^)	
	Surgical	Contralateral	Surgical	Contralateral
ST	8.8 ± 3.6	11.4 ± 3.3	114.8 ± 67.6	214.9 ± 70.4
GR	4.5 ± 1.8	6.3 ± 2.6	69.6 ± 38.8	107.6 ± 44

Cross sectional area (CSA) and volume (mean ± standard deviation) (N = 28) pooled from Williams et al. (2004) (n = 8) and Konrath et al. (2016) (n = 20) for the ST/GR of the Surgical and Contralateral limb.

Using the *V*_*m*_ and *CSA*_*m*_ changes to the ST and GR, the ($l_{m}^{o}$), ($l_{t}^{s}$) and strength coefficients were adjusted. Physiological cross sectional area (*PCSA*_*m*_) is a commonly used muscle parameter, but was not reported, so we assumed *PCSA*_*m*_ = *CSA*_*m*_. Therefore, the *CSA*_*m*_ of an MTU is directly proportional to the strength coefficient (SC_m_) multiplied by ($F_{m}^{MAX}$), from which we develop Eq 1.

$$\frac{{CSA}_{m}^{Surg}}{{CSA}_{m}^{Con}}=\frac{{SC}_{m}^{Adj}}{{SC}_{m}^{Norm}}$$ (Eq 1)

Where *CSA*^*Surg*^ and *CSA*^*Con*^ represent the average *CSA* of the muscles of the surgical leg and contralateral leg in ACLR patients respectively, while ${SC}_{m}^{Adj}$ and ${SC}_{m}^{Norm}$ represent the strength coefficients for the adjusted model and standard model. From this we develop values of (${SC}_{m}^{Adj}$) for the ST and GR.

Volume (*V*_*m*_) of a muscle is related to its cross sectional area (*CSA*_*m*_) multiplied by optimal muscle fibre length ($l_{m}^{o}$). Therefore $l_{m}^{o\;Adj}$ can be approximated, assuming $\emptyset_{m}^{o}$ is the same in the surgical and normal contralateral legs, using Eq 2

$$l_{m}^{o\;Adj}=(l_{m}^{o\;Con})(\frac{V_{m}^{Surg}}{V_{m}^{Con}})\frac{1}{\left(\frac{{CSA}_{m}^{Surg}}{{CSA}_{m}^{Con}}\right)}$$ (Eq 2)

Where $l_{m}^{o\;Con}$ represents the contralateral optimal fibre length respectively, while $\left(\frac{V_{m}^{Surg}}{V_{m}^{Con}}\right)$ and $\left(\frac{{CSA}_{m}^{Surg}}{{CSA}_{m}^{Con}}\right)$ represent the ratios between surgical and contralateral legs *V*_*m*_ and *CSA*_*m*_ respectively. Using the new $l_{m}^{o\;Adj}$, the adjusted tendon slack length was calculated using the same optimization method described in the morphometric scaling in which the dimensionless muscle fibre and tendon operating curves were preserved while respecting the MTU length across a range of lower limb joint angles [36].

The functional calibration was then repeated, however, the adjusted $l_{m}^{o}$ ($l_{m}^{o\;Adj}$), adjusted $l_{t}^{s}$ and adjusted strength coefficients for the ST and GR were not allowed to change. Following this calibration, new parameters were calibrated for the other 32 MTUs within the model, as well as the adjusted parameters for the ST and GR representing their morbidity. The *open-loop* prediction system was then run to obtain MTU forces and moments for each of the 34 MTUs in the adjusted model.

### Tibiofemoral joint contact model

**Fig 1:**
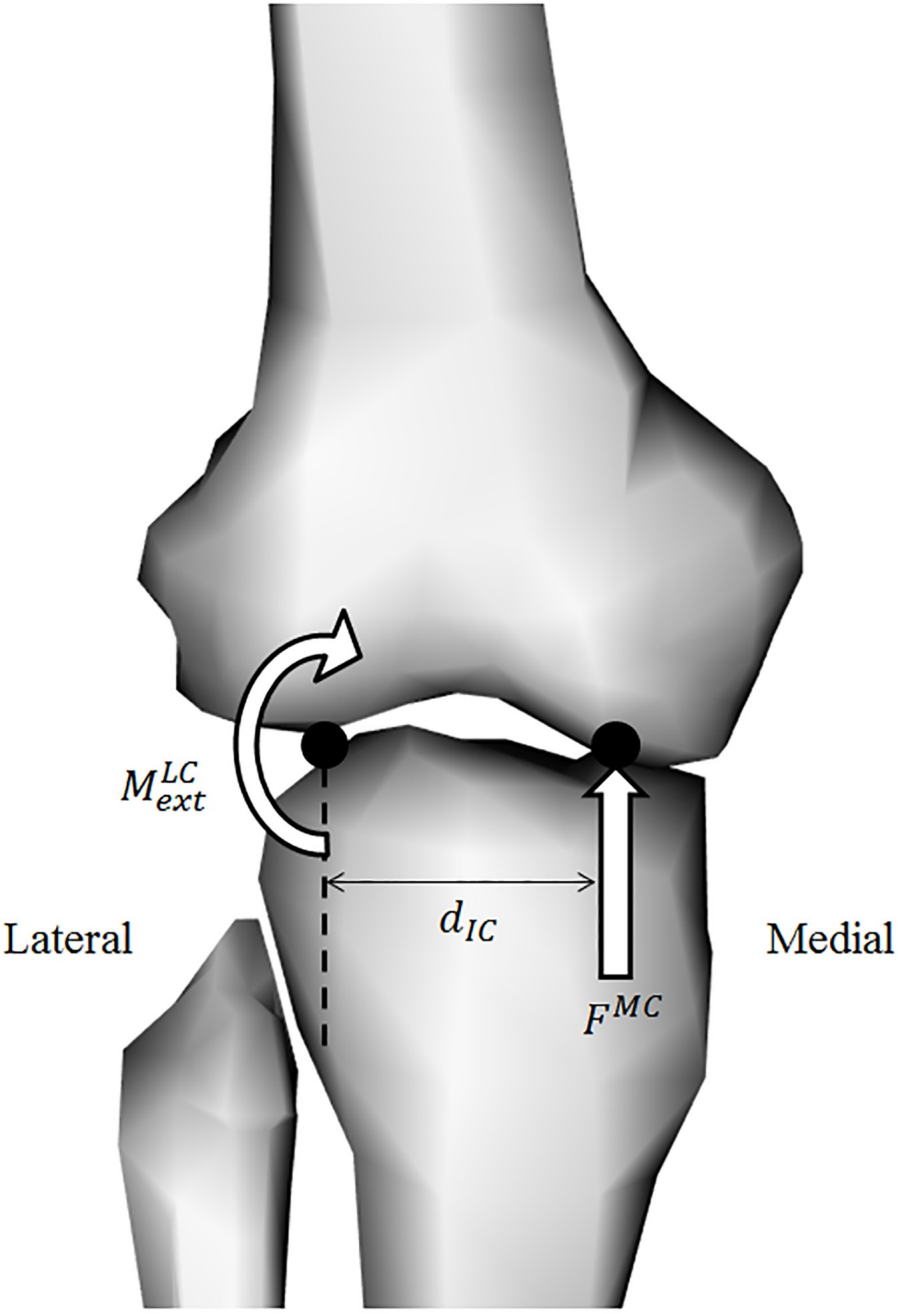
Tibiofemoral joint contact model. Tibio-femoral joint contact model (right leg) used to estimate medial compartment loads (*F*^*MC*^). The patella is not shown. Net moments about the lateral tibial contact point ($M_{MTU}^{LC}+M_{ext}^{LC}$) were divided by the intercondylar distance (*d*_*IC*_).

MTU force estimates from the standard and adjusted model were incorporated into a tibiofemoral joint contact model [10, 22] to estimate the contact load in the medial compartment (F^MC^) (Fig 1). The contact model was based on three assumptions: (i) only forces with a component parallel to the long axis of the tibia or that generate a varus/valgus moment about the knee joint contribute to articular loading, (ii) these loads act through only a single contact point on each condyle, separated by distance (d_IC_), (iii) ligaments do not contribute to loading of the articular surfaces. The net internal MTU varus/valgus moments ($M_{MTU}^{LC}$) about the lateral contact points are first calculated by summing the product of each MTUs force (*F*_*MTU*_) multiplied by its varus/valgus moment arm ($r_{MTU}^{LC}$) about the lateral condyle for *n* MTUs, using Eq 3.

$$M_{MTU}^{LC}=\sum_{i=1}^{n}F_{MTU}(i)r_{MTU}^{LC}(i)$$ (Eq 3)

The difference between $M_{MTU}^{LC}$ and the external moments about the lateral tibial contact points ($M_{ext}^{LC}$) can be used with the intercondylar distance (*d*_*IC*_) to calculate the medial condyle contact force (F^MC^), by assuming static equilibrium about the lateral tibial contact point in the frontal plane (Fig 1), using Eq 4. The net internal and external moments about the lateral condyle were then used to establish each muscle’s contribution to the total medial compartment load expressed as a percentage.

$$F^{MC}=\frac{M_{MTU}^{LC}+M_{ext}^{LC}}{d_{IC}}$$ (Eq 4)

### Statistical analysis

A repeated measures general linear model (GLM) was used to assess the effect of model (standard versus adjusted) on each individual knee muscle’s average contribution over stance to the medial compartment load for each gait task. The effect of model on the optimal fibre length, tendon slack length, strength coefficient and shape factor for each muscle was also tested using the same repeated measured GLM. All statistical analysis was performed using SPSS version 22 (SPSS Inc, Chicago, Ill). Significance was accepted for p<0.05, but to account for multiple GLM comparisons, Benjamini and Hochberg corrections were applied [37].

## Results

**Fig 2:**
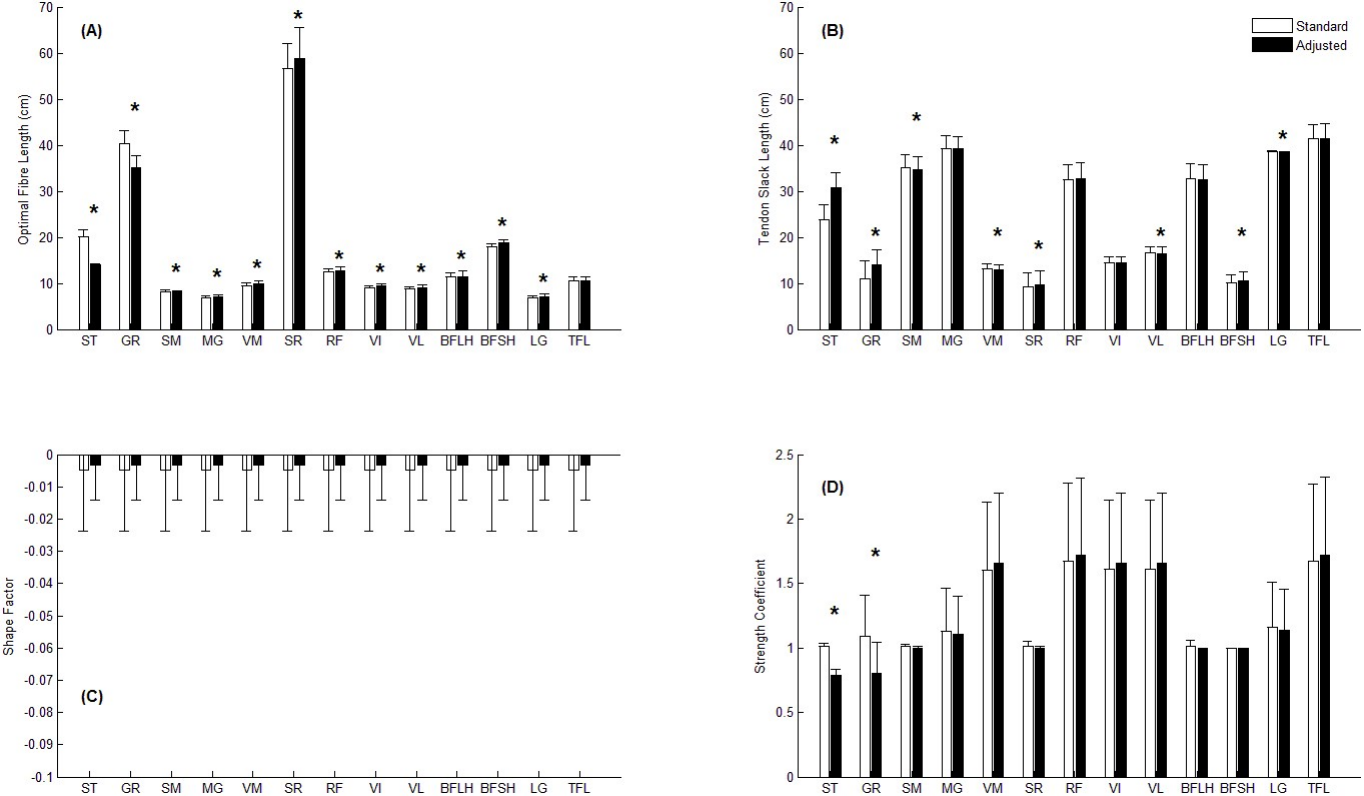
Muscle parameter changes. (A) Optimal fibre length, (B) Tendon slack length, (C) Shape factor and (D) Strength coefficient for each knee muscle in the standard (white) and adjusted (black) model. Data are expressed as mean ± one standard deviation. (*) denotes statistical significance.

ST and GR had significantly shorter optimal fibre lengths, longer tendon slack lengths and reduced strength coefficients in the adjusted compared to standard model (Fig 2). For the medial non-donor muscles, SM, MG, VM and SR significantly increased their optimal fibre length, the SM and VM significantly decreased their tendon slack length, while the SR significantly increased tendon slack length in the adjusted model. For the lateral non donor muscles, RF, VI, VL, BFSH and LG all significantly increased optimal fibre length, VL and LG decreased their tendon slack length, while the BFSH increased its tendon slack length in the adjusted model.

**Fig 3:**
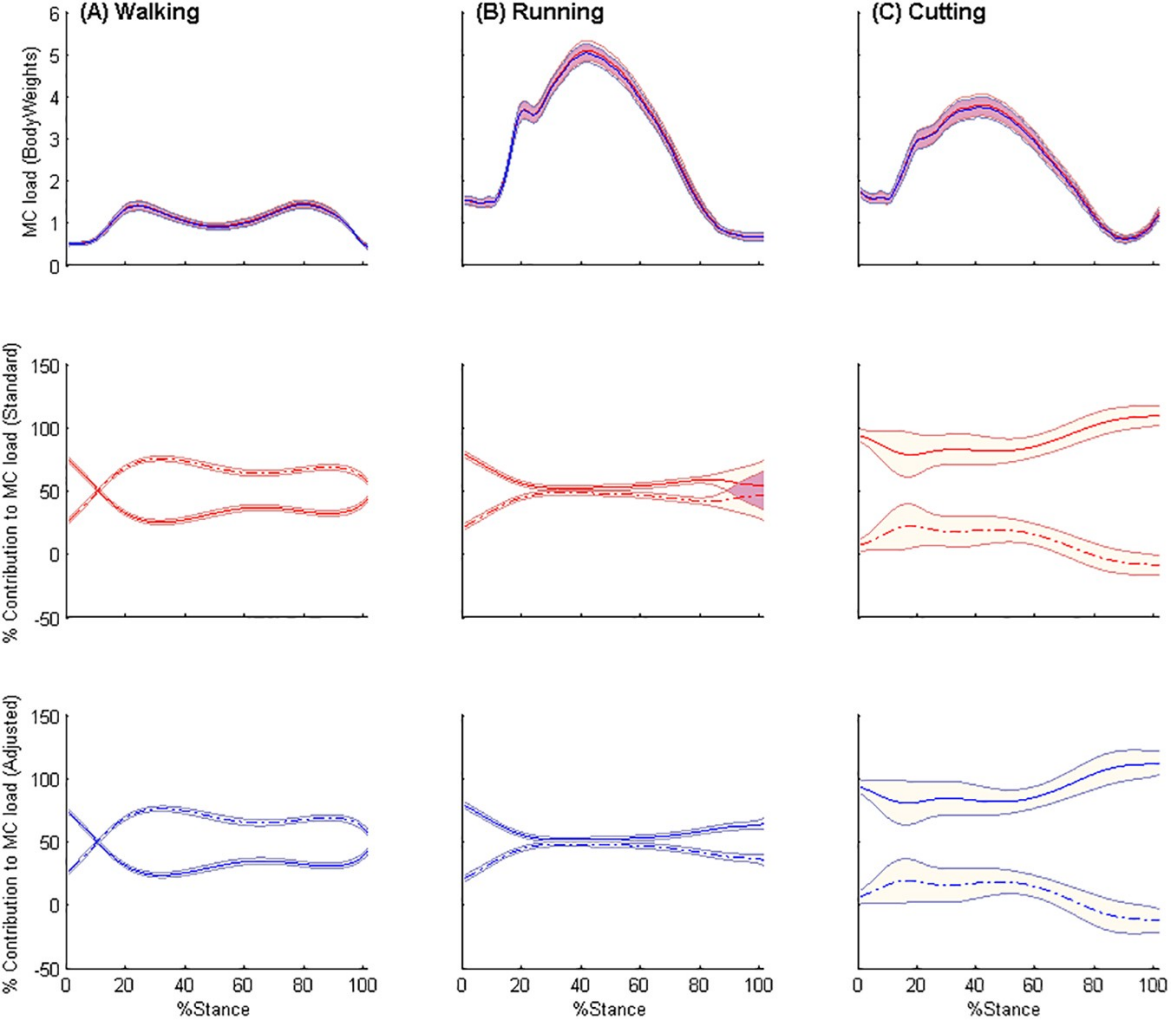
Medial compartment contact loads and relative contributions. Medial compartment (MC) load (Bodyweights), and relative contribution of net internal (solid lines) and external moments (dashed lines) to the MC load (%) for standard (red) and adjusted (blue) models for (A) walking (B) running and (C) cutting. Shaded regions indicate ± one standard error.

The standard and adjusted model produced near identical estimates of the medial compartment tibiofemoral joint contact loads as well as the relative contributions of the internal (MTUs) and external moments to the medial compartment tibiofemoral joint contact load for each gait task (Fig 3). There were no significant differences in either the peak or average medial compartment tibiofemoral joint contact loads between the standard and adjusted model. Similarly, there were no significant differences between the external and internal contributions to the medial compartment tibiofemoral joint contact loads between the standard and adjusted model. Medial compartment loads were lowest in walking and highest in running. External moments were the major contributors to the medial compartment joint contact load in walking whereas the internal moments were the major contributors during running and cutting (Fig 3).

**Fig 4:**
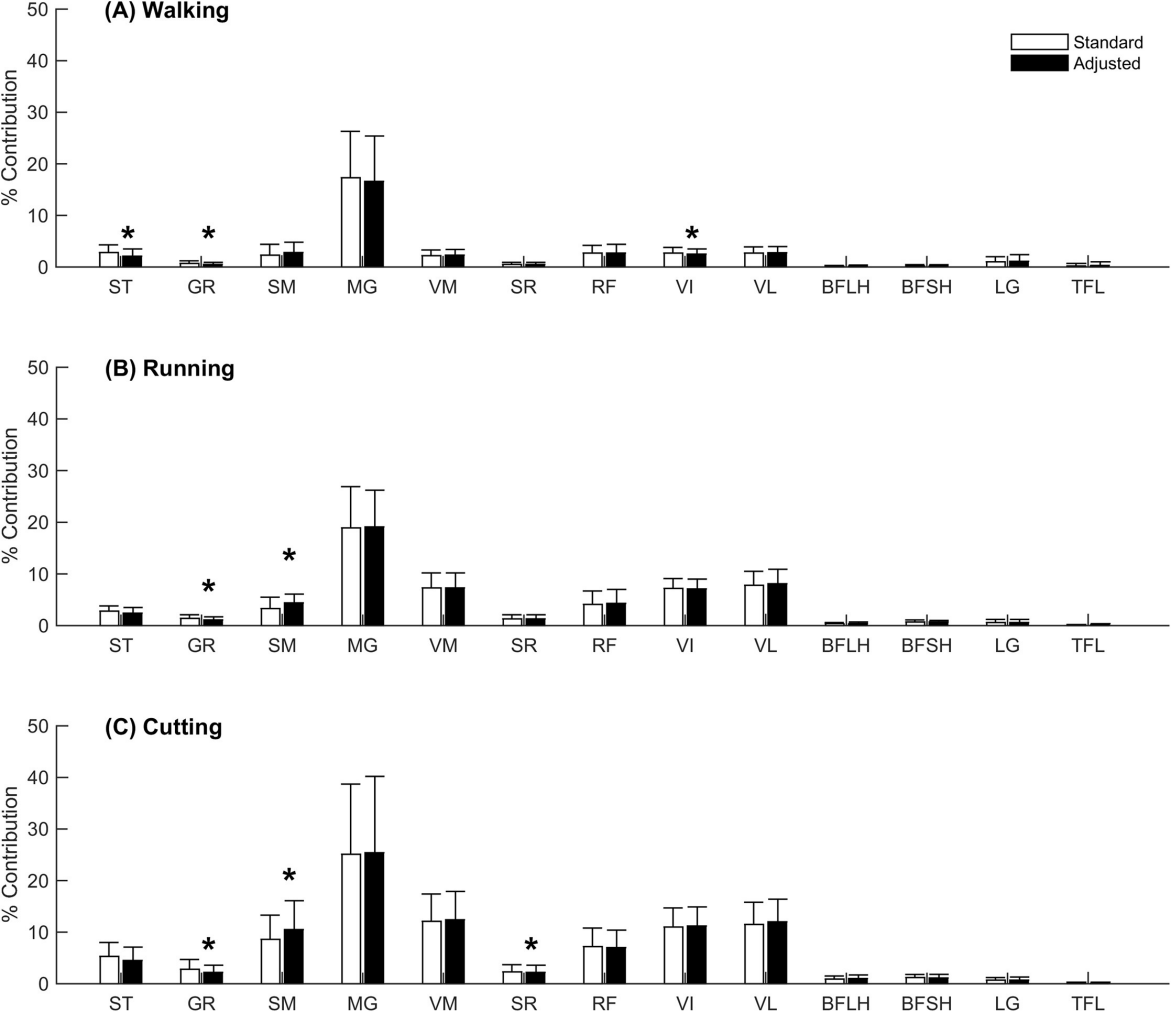
Muscle contributions to medial compartment contact loads averaged over stance. Muscle contributions to the total medial compartment load averaged over stance phase for the standard (white) and adjusted (black) models during (A) walking, (B) running and (C) cutting. Error bars represent ± one standard deviation. (*) denotes statistical significance.

The combined contributions of ST and GR to medial compartment load in the adjusted model were reduced by 26%, 17% and 17% during walking, running and cutting, and were primarily offset by corresponding increases in the SM contributions of 33% and 22% during running and cutting (Fig 4) respectively, however no increase in SM contributions were observed during walking.

During walking, the average contribution of ST, GR and VI to the medial compartment load throughout stance were significantly reduced in the adjusted versus standard model (Fig 4). During running, the contribution of GR, was significantly reduced in the adjusted versus standard model, whereas the contribution of SM was significantly increased (Fig 4). During cutting, the contributions of GR and SR were significantly reduced in the adjusted versus standard model, whereas the contribution of SM was significantly increased (Fig 4).

## Discussion

The CSA and volume of the ST and GR have been reported to be substantially reduced in the surgical leg of ACLR patients compared to the contralateral leg [5, 6]. This study used a computational modelling approach to investigate the theoretical effect of the loss of donor muscle size following a hamstring autograft ACLR, on the medial tibiofemoral compartment contact loads experienced under the same motion and external loading conditions, during walking, running and cutting. The combined contributions of ST and GR to medial compartment tibiofemoral contact loads from our neuromusculoskeletal model were reduced between 17% and 26% across gait tasks. In compensation for the loss of donor muscle size, the SM, in particular, increased its contribution to the medial compartment tibiofemoral contact load. Given that the donor muscles are involved in several degrees of freedom, these findings raise the question of whether post-ACLR rehabilitation programs could be designed to facilitate compensation for donor muscle morbidity by non-donor muscles.

### Muscle-tendon parameters and medial compartment tibiofemoral contact loads in the adjusted versus standard model

The reductions in muscle CSA and volume for the donor muscles that were implemented in the adjusted model were 33% and 47% for the ST, respectively, and 35% and 39% for the GR, respectively [5, 6]. Consequently, the ST and GR muscles were given 30% and 13% shorter optimal fibre lengths, 30% and 27% longer tendon slack lengths, and 22% and 26% smaller strength coefficients respectively. The combined effect of these changes was a decreased force producing capacity of the ST and GR, driven by smaller muscle fibres operating at shorter lengths at faster shortening speeds. In contrast, the SM experienced a 2.3% increase in optimal fibre length and a 1.1% reduction in tendon slack length, which enhanced the force producing capacity of the SM in the adjusted model. The decreased forces from the ST and GR would have reduced the predicted moments about the knee flexion/extension, hip flexion/extension and hip abduction/adduction degrees of freedom. However, the predicted moments of the adjusted model were required to closely match the experimental moments, therefore compensation was needed for the loss of ST and GR strength. These losses in combination with the demand for matching external torques may explain what drove the compensatory changes to the SM, due to its close proximity to the morbid donor muscles and its capacity to exert moments at the knee.

As a result of the abovementioned compensatory changes in muscle-tendon parameters in the adjusted compared to the standard model, there were no significant differences in the overall medial compartment tibiofemoral contact loads between models across the different gait tasks. Furthermore, the relative contribution of the internal and external moments to the medial compartment contact load for each gait task were not different between models. The medial compartment contact loads experienced during walking showed the typical double-peak pattern reported in previous studies [9, 10], with a slightly greater contribution from external moments compared to the net internal moments. Winby and colleagues [10] reported an approximately equal contribution from external and net internal moments to the medial compartment loads during walking in healthy controls, which suggests ACLR patients may have lower muscular contributions to articular loading than healthy controls during walking. The present study also demonstrated the net internal moments to be the major contributor to medial compartment loading during running and cutting, with the net internal moments contributing greater than 100% of medial compartment loads during the late stages of cutting (see Fig 3). The external moments have a negative contribution to the medial compartment load during cutting, as they act to unload the medial compartment. Thus, the internal moments from MTUs are dominant at this time to provide stability and prevent condylar lift-off.

### Contributions of donor muscles to the medial compartment tibiofemoral contact loads in the standard and adjusted model

**Fig 5:**
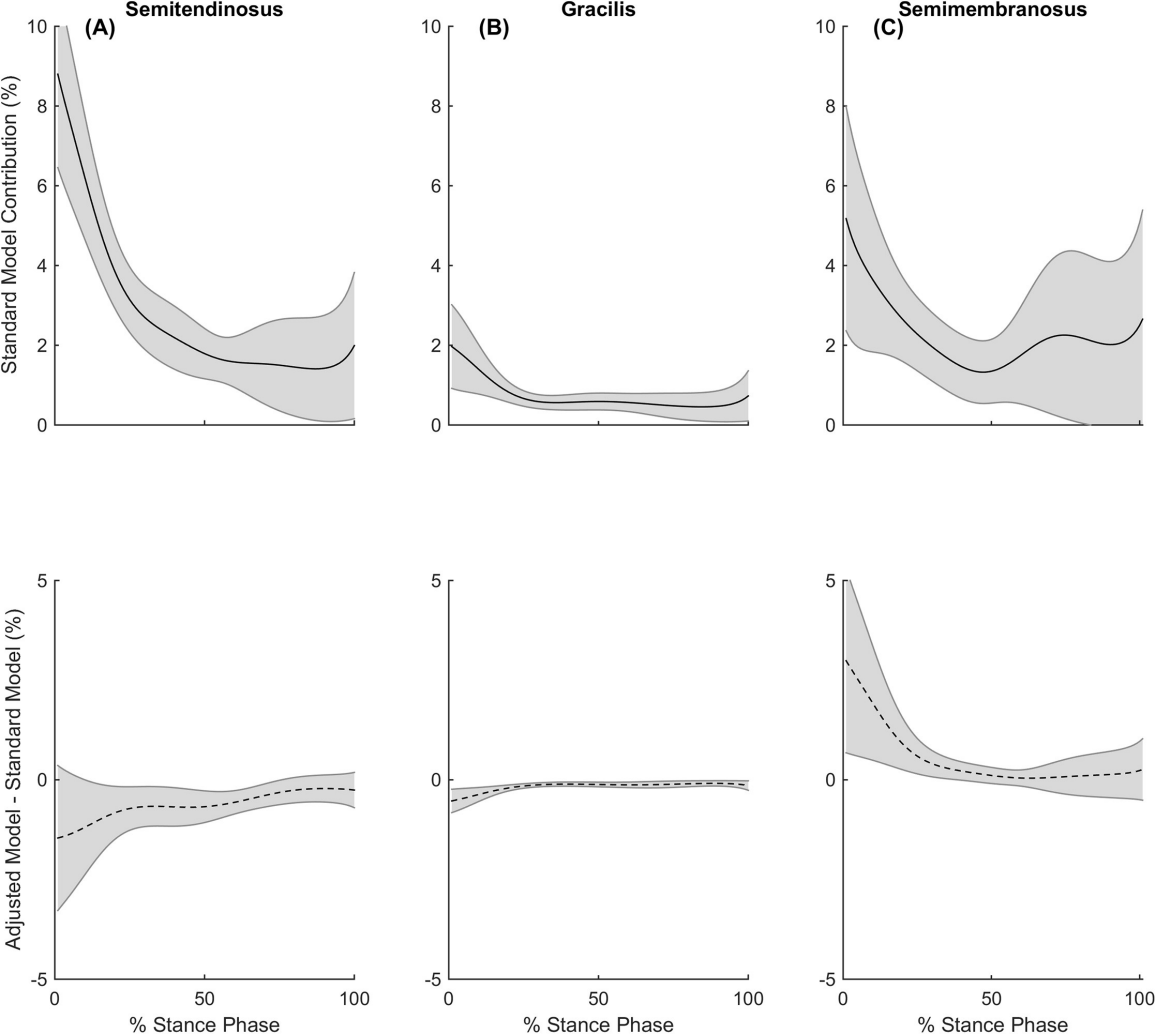
Muscle contributions to medial compartment contact loads during walking. During stance phase of walking, the standard model’s contribution to the medial compartment contact load (solid line) and the contribution differences between standard and adjusted models (dashed line) for the (A) semitendinosus (B) gracilis and (C) semimembranosus. Shaded regions indicate ± 95% confidence interval.

The combined contributions of ST and GR to the medial compartment contact load were reduced during all gait tasks in the adjusted model. During walking, the ST and GR were observed to reduce their contribution to the medial compartment contact load by an average of 25% and 29% over stance, respectively, with the deficits occurring mainly during early stance (Fig 5). Previous modelling studies have investigated the muscular contributions to joint contact forces in healthy controls [9, 10, 38] and found the quadriceps and hamstring muscles dominate the muscular contributions to medial compartment contact loading during early to mid-stance, with the gastrocnemii contributing the majority during late stance. This dominance of the gastrocnemii over the hamstrings during late stance may explain the small reductions in ST and GR contributions to medial compartment contact loading due to their altered morphology (see Fig 5).

**Fig 6:**
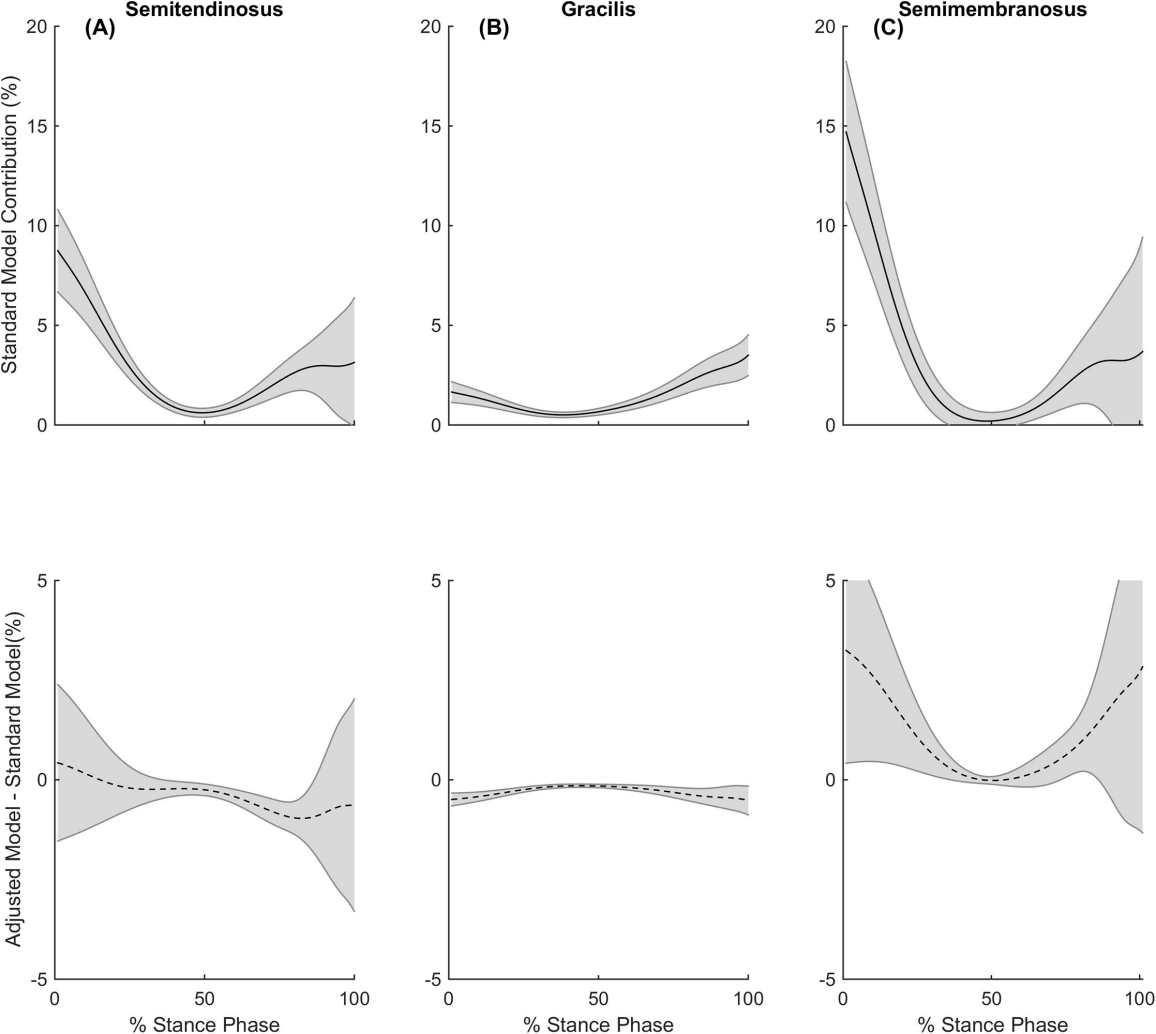
Muscle contributions to medial compartment contact loads during running. During stance phase of running, the standard model’s contribution to the medial compartment contact load (solid line) and the contribution differences between standard and adjusted models (dashed line) for the (A) semitendinosus (B) gracilis and (C) semimembranosus. Shaded regions indicate ± 95% confidence interval.

During running, the GR was observed to reduce its contribution to the medial compartment contact load by an average of 24% over stance and occurred during both early and late phase of stance, with deficits also being seen from the ST during late stance (Fig 6). Previous studies [39, 40] have suggested the quadriceps contribute to braking force during early stance, with the hamstrings playing a major role in the contribution to forward propulsion. Hamner and colleagues [41] reported the quadriceps to be a large contributor to braking force in the early stance phase in running, with the plantar flexors and hamstrings contributing to propulsion, while Sasaki and colleagues [40] found in running, the plantar flexors work in synergy with hip and knee extensors near mid stance to provide forward propulsion. Collectively, these observations may explain why differences can be seen in the ST and GR contributions to the medial compartment contact load during the mid to late phase of stance of running.

**Fig 7:**
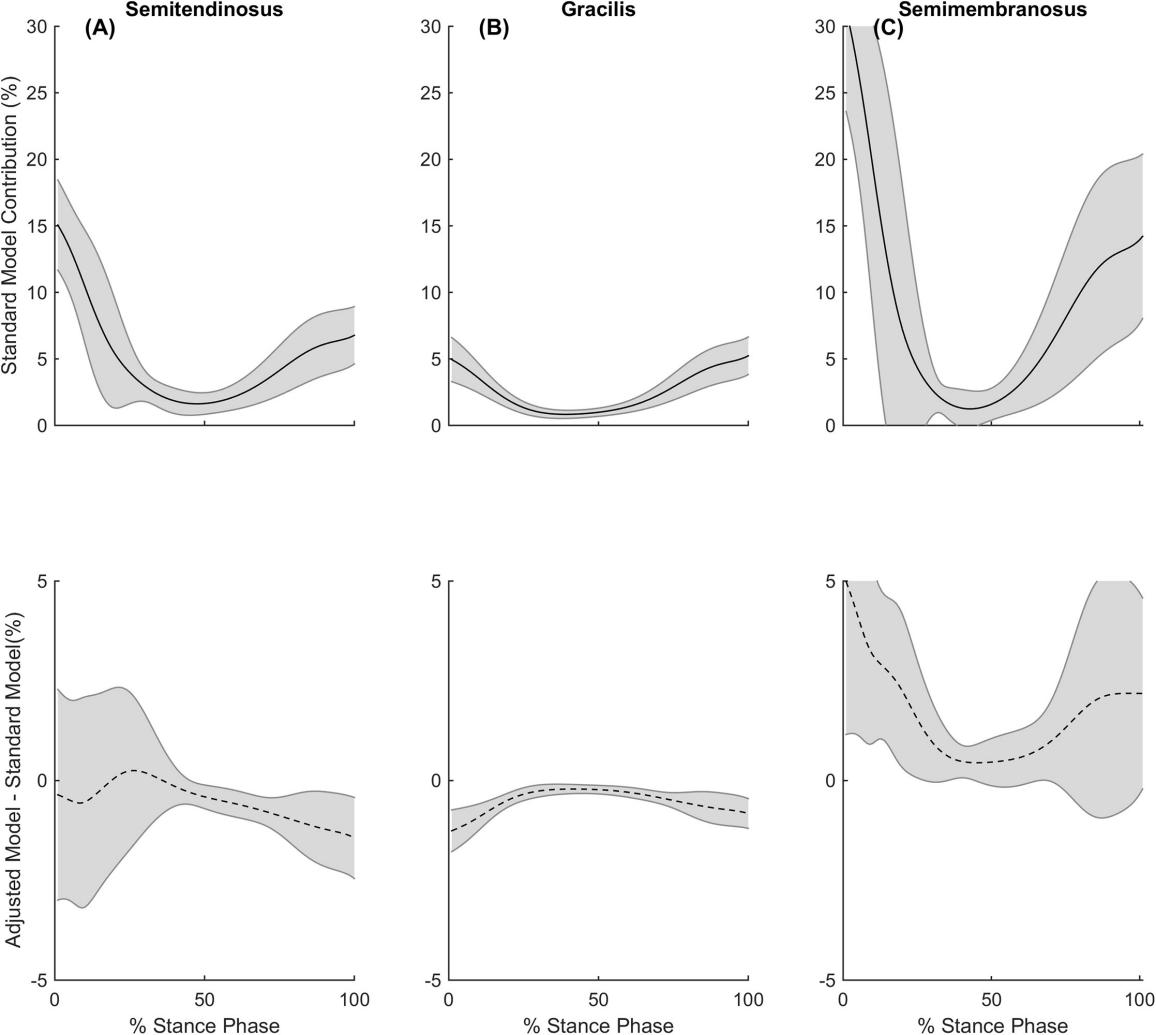
Muscle contributions to medial compartment contact loads during cutting. During stance phase of cutting, the standard model’s contribution to the medial compartment contact load (solid line) and the adjusted model difference (dashed line) for the (A) semitendinosus (B) gracilis and (C) semimembranosus. Shaded regions indicate ± 95% confidence interval.

Whilst performing sidestep cutting manoeuvers, the GR contributed on average 21% less to the medial compartment contact load during stance, with the differences occurring over the entire stance phase. Previous studies investigating the muscle activation strategies at the knee during sidestep cutting manoeuvers have found the activation of medial muscles including ST, GR, SM and MG in order to stabilise the external valgus moments that occur [42, 43], this selected activation is believed to directly stabilise the external moment preventing condylar lift off [11, 44]. This may explain why we saw deficits over the whole stance phase for GR, and also during late stance for ST (Fig 7).

### Contributions of non-donor muscles to the medial compartment tibiofemoral contact loads in the standard and adjusted model

The contribution of SM to the medial compartment contact load increased by 33% and 22% during running and cutting respectively. The increased contributions also appear to be in similar phases of stance to the deficits that were observed from the ST and GR for each respective gait task. The SM increased its contributions during the early stance phase of walking, likely due to the hamstrings involvement in early stance [9, 10, 38] (Fig 5). The SM also increased its contribution during the early and late stance phase of running, likely due to the hamstrings involvement in propulsion [39–41] (Fig 6). Furthermore, the SM increased its contribution to medial compartment contact loading during cutting, likely due to their selected activation to stabilise external valgus moments and generate forward propulsion [42, 43] (Fig 7).

This study has demonstrated theoretically that non-donor muscles including the SM are able to compensate for donor muscle morbidity, by stabilising the tibiofemoral joint in the frontal plane during demanding and complex locomotor tasks. The extent to which this occurs in practice is unclear, but it has been reported that SM and BFLH muscle volume of the surgical limb are higher than for the contralateral limb at two years following a quadruple bundle hamstring graft in ACL reconstruction [6]. This suggests that a degree of compensation for donor muscle morbidity does occur, which is consistent with modelling results in the present study, since the optimal fibre lengths increased for the SM. The extent to which this compensation effect can be amplified by targeted rehabilitation warrants some further consideration. This may have important implications for the future health of the tibiofemoral joint following ACLR, since there is emerging evidence that loading of the medial compartment in ACLR patients using hamstring grafts may be lower than normal, and related to the early onset of knee OA [15].

The present study suggests that under-loading of the medial tibiofemoral joint compartment may not be due to atrophy of the donor muscle, as other muscles have the potential to take over the role of the atrophied ST and GR. This is also reflected in animal models, in which loss of muscle strength via nerve setting or botox injections is associated with compensatory hypertrophy of muscles with similar function [45]. However under-loading is still an issue that needs to be addressed if we are to ameliorate the potential development of knee osteoarthritis in ST-GR ACLR populations. Future research should further investigate the neuromuscular biomechanical factors that contribute to lower joint contact forces. Recent literature has shown that lower knee extension moments may be a potential contributor to lower joint contact forces [18], and have suggested increased quadriceps strength or promoting a gait pattern that doesn’t have a knee extension moment avoidance strategy.

### Limitations

Subject-specific data was not used to represent each participant’s morphological changes to the ST and GR, but rather an average from what has already been established in the literature [5, 6]. In addition, peak CSA from the literature was used rather than physiological CSA, however, the pennation angle is quite small in the ST and GR, so the differences would be considered negligible. Another point to address is that imaging modalities were not used for determining the location of the medial and lateral tibial contact points, but rather a regression function following morphometric scaling. This may have had a small effect on the moment arm calculations of the muscles with respect to the medial and lateral condyles. A final limitation of the present study is that we did not have an ACL reconstructed cohort of different graft types that may provide comparisons to show the relative effects of the different surgeries on tibiofemoral joint contact forces.

## Conclusion

The contribution of ST and GR muscle forces to medial compartment tibiofemoral contact loading during walking, running and cutting manoeuvers is reduced following ACLR with the use of a hamstring graft. However, it appears possible that non-donor muscles, most notably the semimembranosus, can compensate for donor muscle morbidity by increasing contributions to the medial compartment tibiofemoral contact loading during the locomotor tasks we investigated. Whether these compensatory adaptations may be achieved in practice through targeted rehabilitation training programs requires investigation.
